# Metals and Metal Complexes in Diseases with a Focus on COVID-19: Facts and Opinions

**DOI:** 10.3390/biology12060868

**Published:** 2023-06-15

**Authors:** Agnieszka Ścibior, Manuel Aureliano, Alvin A. Holder, Juan Llopis

**Affiliations:** 1 Laboratory of Oxidative Stress, Department of Biomedicine and Environmental Research, The John Paul II Catholic University of Lublin, 20-708 Lublin, Poland; 2 Faculdade de Ciências e Tecnologia (FCT), Campus de Gambelas, Universidade do Algarve, 8005-139 Faro, Portugal; 3 Centro de Ciências do Mar (CCMar), Campus de Gambelas, 8005-139 Faro, Portugal; 4 Department of Chemistry and Biochemistry, Old Dominion University, Norfolk, VA 23529, USA; aholder@odu.edu; 5 Institute of Nutrition and Food Technology “José Mataix Verdú”, Department of Physiology, Biomedical Research Center, University of Granada, Avda del Conocimiento sn., 18100 Armilla, Granada, Spain; jllopis@ugr.es

## 1. Introduction and Scope

**Figure 1:**
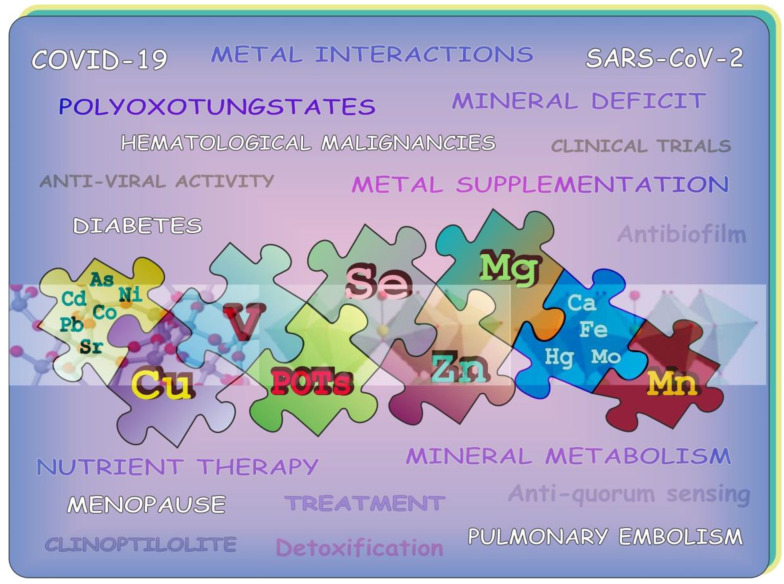
Graphical summary of metals, some metal cluster anions, and certain natural minerals overviewed in this Special Issue: biological applications, diseases, detoxification, antibiofilm formation, among others.

In the present Special Issue on “Metals and Metal Complexes in Diseases with a Focus on COVID-19: Facts and Opinions”, an attempt has been made to include reports updating our knowledge of elements considered to be potential candidates for therapeutic applications and certain metal-containing species, which are extensively being examined towards their potential biomedical use due to their specific physicochemical properties. The Special Issue compiles data on the role of metals in COVID-19 and focuses on other illnesses and biological processes that affect metal metabolism. It consists of eight manuscripts, including five review articles and three original research papers (Figure 1). These papers garnered (June 2023) a total of 12 citations and above 10,500 visualizations.

Further strategies to improve the pharmacological features of metals and metal-containing compounds outside of COVID-19 are intensively studied, with a special focus on the susceptibility of different cells to their effects, key ligands affecting the mode of their action, and the correlations between biological effects of metals/metal-containing complexes and potential therapies for human pathologies [1,2,3,4,5,6,7]. 

## 2. Contributions

The first paper published in the present Special Issue (SI) is entitled “Elements and COVID-19: A Comprehensive Overview of Studies on Their Blood/Urinary Levels and Supplementation with an Update on Clinical Trials” [8]. The Ścibior research group is particularly recognized for its significant contributions to the field of vanadium studies in biology and toxicology [5,9]. The review article by Ścibior and Wnuk overviews the levels of some metals in the blood and urine of COVID-19 patients, summarizes findings on supplementation with certain micronutrients in COVID-19 subjects, and provides an update on clinical trials aimed to examine the effects of administration of minerals, alone or in combination with other agents in COVID-19 patients and COVID-19 risk groups. The data compiled in this review may lay the groundwork for new therapeutic treatments and further research on biomarkers of the COVID-19 course and prognosis. The authors highlight the need for clarification of the importance of mineral supplementation in the COVID-19 course and the relationships of the levels of some elements with clinical improvement [8].

In turn, a review conducted by Szewc et al. collected information about the role of certain minerals, i.e., zinc (Zn) and copper (Cu), in platelet activation and pathophysiological thrombus formation in patients with pulmonary embolism in the course of SARS-CoV-2 infection [10]. The authors graphically portrayed (a) the process of platelet activation caused by Zn, (b) the course of SARS-CoV-2 infection and possible options for the pathway of the disease process, and (c) the risk factors predisposing populations to pulmonary embolism. As highlighted in the paper, it is important to understand the exact pathophysiological mechanism of thrombus formation in patients with pulmonary embolism, as it is one of the most common complications in the course of SARS-CoV-2 disease. The authors also indicated the need for exploring the therapeutic options of Zn and Cu in the course of COVID-19 thrombus formation with an emphasis on the dosage and duration of their misbalance [10].

The next review, conducted by the Męcik-Kronenberg group, focused on metal interactions, menopause, and COVID-19 [11]. The authors concisely described the picture of menopause, provided a discussion on the potential impact of menopause on the course of COVID-19, summarized recent findings on the relationship between the exposure to some metals and menopause, and collected information about supplementation with certain elements in menopausal woman and the relationship between metals and SARS-CoV-2 infection in humans. They emphasized the need for supplementation with some minerals in menopausal woman to alleviate the clinical symptoms of COVID-19 and the toxic effects of metals, they and drew attention to the need for further larger-scale studies on the effects of metals on the course of menopause [11].

A review written by several members of the MaGNet Global Magnesium Project was devoted to the importance of magnesium (Mg) status in COVID-19 [12]. It collected information on the serum Mg level in COVID-19 studies, dietary Mg intake in the COVID-19 course, Mg supplementation in COVID-19 patients, mechanisms of Mg action in pulmonary complications of COVID-19, and implications of Mg in the pathogenesis and evolution of certain CNS complications in COVID-19. The potential of Zn and/or Mg as useful agents in increasing the effectiveness of drug therapy or reducing the adverse effects of anti-COVID-19 drugs was also discussed. Simultaneously, the authors highlighted the need for studies on implications of the disruption of normal or optimal dietary and circulating ratios between Mg and other bivalent cations, such as Zn and Cu, as well as certain vitamins, e.g., vitamin D, important in patients with COVID-19. They also stressed the need for more clinical trials focused on the therapeutic effects of combining Mg and Zn with medications used to fight against COVID-19 and for studies on the efficiency of different compounds with Mg and Zn used in conjunction with anti-COVID-19 medication, i.e., compounds with different pharmacokinetic parameters [12].

The last review, written by Hus et al., was devoted to COVID-19 in adult patients with hematological malignancies (HMs). In this paper [13], the authors listed the causes of the higher susceptibility of patients with HM to infection complications and summarized the structure and variants of SARS-CoV-2 and the evolution of the COVID-19 clinical course in patients with hematological neoplasms along with virus mutations. They also collected data on the treatment of COVID-19 in patients with HM and on the COVID-19 vaccines approved in the European Union that can be used both as primary vaccination and booster together with their clinical efficacy in cancer patients. Additionally, they graphically illustrated the history of the most important drugs (registered by the European Medicines Agency) that are currently used for the treatment of this disease and discussed the effects of COVID-19 in patients with HMs before and after COVID-19 vaccination. Finally, the authors referred to supplementation with one of the minerals that could be potentially considered in COVID-19 patients with HMs as adjunct therapy and provided current recommendations for prophylactic and therapeutic management in HM patients [13].

The original articles focused on (1) the assessment of the potential biomedical use of polyoxotungstates (POTs) [14], (2) the influence of vanadium (V) on the homeostasis of some minerals and divalent metal transporter 1 (DMT1) mRNA expression in streptozotocin-induced hyperglycemic rats [15], and (3) the in vivo efficiency and safety of long-term administration of clinoptilolite in its activated forms [16], i.e., tribomechanically activated zeolite (TMAZ) and panaceo micro-activated zeolite (PMAZ) on the biodistribution of such metals as Al, As, Cd, Co, Pb, Ni, and Sr.

Aureliano’s research group, in collaboration with other scientists, conducted a series of in vitro and in vivo studies focused on vanadium compounds, particularly the polyoxovanadate decavanadate ([V_10_O_28_]^6−^, V_10_). The primary objective of these investigations was to evaluate the potentially toxic effects of V_10_ [6], as well as the interactions with proteins [17]. Furthermore, the research aims to explore the anticancer, antibacterial, and antidiabetic properties of polyoxovanadates (POVs), both individually [6,7,17] and in combination with metformin, specifically in relation to human melanoma cells [18]. The data reported by Faleiro and co-workers on another type of polyoxometalate (POM), this time a polyoxotungstate (POT) [14], show that, in addition to antibacterial activity, the Preyssler-type POT P_5_W_30_ is able to impair quorum sensing and biofilm formation, which facilitate bacterial colonization, antibiotic resistance, and persistence in both the environment and the host. Additionally, P_5_W_30_ has been demonstrated to exhibit anti-viral activity against enteric viruses [14].

The results obtained by Sánchez-González et al. [15] show that diabetes alters the metabolism of such minerals as Zn and Cu and, to a lesser degree, Mn, and that bis(maltolato)oxovanadium (IV), BMOV, administered to diabetic rats as a hypoglycemic agent at a dose of 3 mg V/day reverts the changes in Zn and Cu homeostasis caused by this disease. The authors suggest that the effect of supplementation with BMOV is possibly mediated by a fall in food intake and DMT1 gene expression. As stressed, more studies are needed to better determine the mode of action responsible for the observed changes, recognize the effects of the interactions, and evaluate the optimum level of pharmacological intervention in order to avoid or prevent side effects [15].

Finally, the findings obtained by Dolanc et al. [16] show a beneficial effect of oral administration of zeolite clinoptilolite materials on the concentration profile of Al, As, Cd, Co, Pb, Ni, and Sr in the blood, brain, bone, kidney, and intestine in healthy female rats. The authors noted a release of the metals from the organs to the bloodstream, which, as they highlight, may point to a detoxification process [16].

## 3. Conclusions and Outlook

There is an increasing interest in the potential applications of metals in medicine. The present Special Issue reflects distinct and emergent biomedical applications of metal compounds and metals in the 21st century, exhibiting, for instance, activities against COVID-19, among other diseases. We believe that the information provided in this Special Issue will be valuable to readers who are interested in metals and metal complexes/metal coordination compounds in general and to those interested in the role of elements in biological processes and diseases, including COVID-19.
